# Circulating levels of matrix metalloproteinase-9 (MMP-9), neutrophil gelatinase-associated lipocalin (NGAL) and their complex MMP-9/NGAL in breast cancer disease

**DOI:** 10.1186/1471-2407-9-390

**Published:** 2009-11-04

**Authors:** Xeni Provatopoulou, Antonia Gounaris, Eleni Kalogera, Flora Zagouri, Ioannis Flessas, Evgenios Goussetis, Afroditi Nonni, Ioannis Papassotiriou, George Zografos

**Affiliations:** 1 Research Center, Hellenic Anticancer Institute, Athens, Greece; 2 Breast Unit, First Department of Propaedeutic Surgery, Hippokratio Hospital, University of Athens, Athens, Greece; 3 Stem Cell Transplant Unit, "Aghia Sophia" Children's Hospital, Athens, Greece; 4 First Department of Pathology, School of Medicine, University of Athens, Athens, Greece; 5 Department of Clinical Biochemistry, "Aghia Sophia" Children's Hospital, Athens, Greece

## Abstract

**Background:**

Recent evidence suggests that neutrophil gelatinase-associated lipocalin (NGAL) expression is induced in many types of human cancer, while detection of its complex with matrix metalloproteinase-9 (MMP-9) is correlated with cancer disease status. We aim to evaluate the serum expression of MMP-9, NGAL and their complex (MMP-9/NGAL) during the diagnostic work-up of women with breast abnormalities and investigate their correlation with disease severity.

**Methods:**

The study included 113 women with non-palpable breast lesions undergoing vacuum-assisted breast biopsy for histological diagnosis, and 30 healthy women, which served as controls. Expression levels of MMP-9, NGAL and their complex MMP-9/NGAL were determined in peripheral blood samples with immunoenzymatic assays.

**Results:**

Women with invasive ductal carcinoma exhibited significantly increased levels of MMP-9, NGAL and MMP-9/NGAL compared to healthy controls (MMP-9: p < 0.003, NGAL: p < 0.008 MMP-9/NGAL: p < 0.01). Significant correlations were observed between MMP-9 and NGAL serum levels and breast disease severity score (r = 0.229, p < 0.006 and r = 0.206, p < 0.01, respectively), whereas a non-significant correlation was found for their complex. MMP-9, NGAL and their complex MMP-9/NGAL levels were not correlated with either Body Mass Index (BMI) or age of patients.

**Conclusion:**

These findings suggest that the serum measurement of MMP-9 and NGAL may be useful in non-invasively monitoring breast cancer progression, while supporting their potential role as early biomarkers of breast disease status.

## Background

The lipocalins constitute a family of small-secreted proteins capable of binding hydrophobic molecules. Their principal function is the transport of lipophilic substances but they also participate in immunomodulation and synthesis of prostaglandins [1]. Lipocalin 2 or neutrophil gelatinase-associated lipocalin (NGAL) is a prominent member of the lipocalin family and was originally identified as a glycoprotein in complex with matrix metalloproteinase-9 (MMP-9) in human neutrophils [2,3].

NGAL is an acute phase protein and its expression is upregulated under diverse conditions [4,5]. It has been extensively investigated as a biomarker for the early diagnosis of acute kidney injury from diverse etiologies [6-9]. Recent evidence suggests that NGAL expression is induced in many types of human cancer, including breast [10,11], gastric [12], esophageal squamous cell [13], colorectal [5], pancreatic [14,15], lung [16] and ovarian cancer [17]. Regarding breast cancer, Stoesz and coworkers originally reported heterogeneous expression of NGAL mRNA and protein levels in breast cancer tissue that significantly correlated with other markers of poor prognosis including estrogen and progesterone receptor-negative status and high proliferation [10]. Bauer et al recently extended these findings to show that immunohistochemical NGAL expression strongly correlated with negative steroid receptor status, Her-2/neu overexpression, poor histologic grade, the presence of lymph node metastasis and high proliferation index. In univariate analysis, NGAL expression was associated with decreased disease-specific and disease-free survival. In multivariate analysis, NGAL remained an independent prognostic marker for disease-free survival. These data suggested that tissue NGAL expression could be a valuable prognostic marker in patients with primary breast cancer [11]. Gene expression profiling studies have also confirmed the correlation between NGAL expression and negative estrogen receptor status [18-21].

It has been established that NGAL forms a complex with matrix metalloproteinase-9, thereby preventing MMP-9 autodegradation and increasing its activity in vitro [22]. MMP-9 plays a critical role in cancer progression, invasion and metastasis in several neoplastic diseases including breast cancer [23]. Since MMP-9 is implicated in both early and late processes of tumor progression through the degradation of the extracellular matrix and basement membranes [24], the question whether NGAL and MMP-9/NGAL complex contributes to tumor progression was raised.

Fernandez et al investigated the role of MMP-9/NGAL complex in breast tumor growth and its presence in the urine of breast cancer patients [25]. Their findings suggested that detection of urinary MMP-9/NGAL complex might represent an independent predictor of disease status. Recently, Smith and coworkers reported significant elevations in MMP-9 and MMP-9/NGAL in brain tumor patients. Their expression correlated with the presence of disease and the response to therapy and could be detected both in tumor tissue and urine samples [26]. An association between MMP-9/NGAL complex and gastric cancer has also been suggested since complex expression in tumor tissue of gastric cancer patients was highly associated with worse survival and was related to the histological and genetic typing of gastric cancer [12]. Moreover, recent studies have suggested an association between MMP-9/NGAL complex expression and abdominal aortic aneurysms as well as osteoarthitis [27,28].

To date, most studies have focused on NGAL and MMP-9/NGAL tissue expression while only a few have investigated the clinical utility of their urinary measurements. NGAL and MMP-9 are stored in specific granules in neutrophils, while MMP-9 is also found in gelatinase granules. They both mainly exist in forms not associated with each other. Due to its large size, it seems unlikely that the MMP-9/NGAL complex can be directly filtered from serum to urine. It has thus been suggested that MMP-9 and NGAL are mainly secreted in blood by neutrophils infiltrating the tumor, and are separately excreted in urine where they subsequently form complexes [22]. Although the detection of NGAL and its complex with MMP-9 in systemic circulation seems reasonable, no studies of NGAL and MMP-9/NGAL in sera are currently available with the exception of two recent publications on coronary artery disease and polycystic ovary syndrome [29,30]. The aim of our study is to evaluate the serum levels of MMP-9, NGAL and MMP-9/NGAL complex in patients with breast abnormalities and investigate their correlation with breast disease severity.

## Methods

The study included 113 women; 35 with sclerosing adenosis, 18 with atypical ductal hyperplasia (ADH), 32 with ductal carcinoma in situ (DCIS), and 28 with invasive breast cancer (IDC), while 30 healthy women served as controls. The mean age of the patients and controls was 52.8 ± 9.7 and 55.0 ± 13.7 years, respectively, whereas their Body Mass Index (BMI) was 26.2 ± 4.7 and 25.9 ± 4.4 kg/m^2^, respectively. Prior to their enrollment, all the participants were evaluated for the absence of metabolic disorders including diabetes, diagnosed inflammatory disease and abnormal liver and kidney function. Healthy controls were confirmed after clinical examination, mammography and serological analysis during their annual breast screening. Peripheral blood samples were obtained from patients before preoperative histological diagnosis of non-palpable lesions by vacuum-assisted breast biopsy (VABB), after overnight fasting. The histopathological data were reported during the statistical analysis, after the blind completion of the assays. Based on the published relative risk of breast benign diseases for cancer, the women were classified into five groups of increasing severity as follows: Healthy controls = Disease Severity Score 1; Sclerosing Adenosis = Disease Severity Score 2; Atypical Ductal Hyperplasia = Disease Severity Score 3; Ductal Carcinoma In Situ = Disease Severity Score 4 and Invasive Breast Cancer = Disease Severity Score 5. The participants were recruited from the Breast Unit, First Department of Propaedeutic Surgery, Athens University Medical School at Hippokratio Hospital between September 2005 and June 2007. The protocol was approved from the Institutional Research Committee and written informed consent was obtained from each patient prior to study entry.

### Sample Collection and Assays

Peripheral venous blood samples were collected between 12:00 a.m. and 14:00 p.m. into separator vacutainers and allowed to clot for 20 to 30 min at room temperature. The samples were centrifuged at 3000 × g for 15 min at 8°C, divided into aliquots and stored at -80°C until being assayed. The determination of serum MMP-9 concentrations was performed in duplicates, on the Luminex-100 IS (Integrated System) Luminex Corporation, Austin, TX, US using the MMP-9 assay kit manufactured by R&D Systems, Minneapolis, MN, US. Multianalyte profiling calibration microspheres for classification and reporter readings, as well as sheath fluid were purchased from Luminex Corporation. The acquired fluorescence data were analyzed with the Luminex 2.3 Version software. All analyses were performed according to the manufacturers' protocols. Serum NGAL and MMP-9/NGAL levels were determined in duplicate by solid phase ELISA techniques (R&D Systems, Minneapolis, MN, US). According to manufacturers, the intra-assay and inter-assay CVs for NGAL range between 3.1 and 4.1% and 5.6 and 7.9%, respectively, and for MMP-9/NGAL range between 2.3 and 4.1% and 5.1 and 7.6%, respectively.

### Statistical Analysis

Data are expressed as mean ± SD. Comparison of analytes within groups was performed using the Student t-test. The correlation coefficient *r *between the parameters tested was computed using least squares regression analysis. The *p *values reported are two tailed. All the statistical procedures were performed using the STATGRAFICS 5.1 for Windows program (Graphic Software System, STATPOINT TECHNOLOGIES, INC. Warrenton, Virginia, US).

## Results

Benign breast diseases, encompassing atypical ductal hyperplasia and sclerosing adenosis, have been associated with varying relative risk for developing cancer for patients with no family history, depending on their histological features [31]. Based on these data, we classified our patients into five groups of increasing disease severity and we evaluated the correlation between the expression of MMP-9, NGAL, and MMP-9/NGAL complex and severity score. Mean serum levels of MMP-9, NGAL and MMP-9/NGAL complex in healthy women and patients with sclerosing adenosis, ADH, DCIS and IDC are presented in Table 1. Patients with invasive carcinoma exhibited significantly increased levels of all three molecules compared to healthy controls (MMP-9: p < 0.003, NGAL: p < 0.008 MMP-9/NGAL: p < 0.01).

**Table 1:** Serum levels of MMP-9, NGAL and MMP-9/NGAL complex.

	Healthy Controls (N = 30)	Sclerosing Adenosis (N = 35)	ADH (N = 18)	DCIS (N = 32)	IDC (N = 28)
**NGAL (ng/ml)**	70.7 ± 17.4	78.3 ± 28.8	79.1 ± 30.6	80.0 ± 22.5	87.4 ± 28.1*
**MMP-9 (ng/ml)**	264.2 ± 128.3	402.0 ± 284.8	326.0 ± 171.8	330.7 ± 165.7	392.6 ± 181.7**
**MMP-9/NGAL (ng/ml)**	39.9 ± 20.9	54.9 ± 41.0	53.9 ± 36.7	49.5 ± 34.1	60.7 ± 42.3***

Serum levels of NGAL, MMP-9 and MMP-9/NGAL complex in healthy controls and patients with Sclerosing adenosis, Atypical ductal hyperplasia (ADH), Ductal carcinoma in situ (DCIS) and Invasive ductal carcinoma (IDC). Data are expressed as mean ± SD. Significantly increased expression is observed for patients with IDC compared to healthy controls (*NGAL: p < 0.008, **MMP-9: p < 0.003, ***MMP-9/NGAL: p < 0.01).

**Figure 1:**
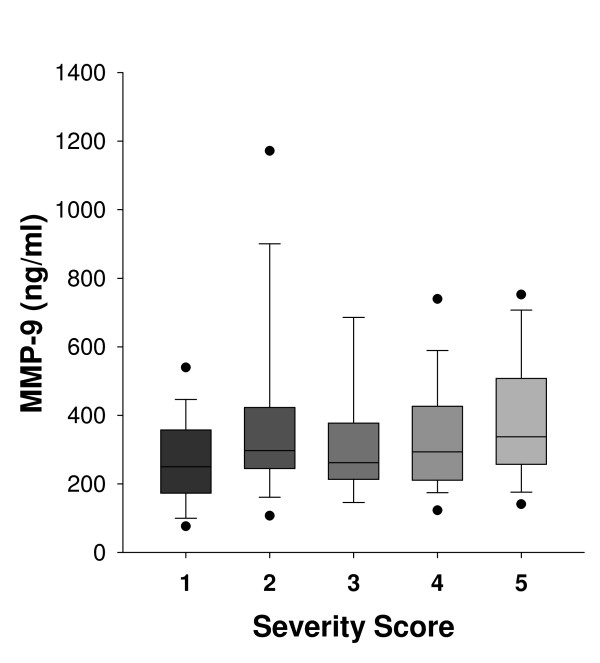
**Serum levels of MMP-9 depicted as box-plots**. (Boxes represent the interquartile range; lines inside boxes represent the median value; whiskers represent 5^th ^and 95^th ^percentiles). The 1-5 scale represents breast abnormalities of increasing severity as follows: 1: Healthy controls (N = 30), 2: Sclerosing adenosis (N = 35), 3: Atypical ductal hyperplasia (ADH) (N = 18), 4: Ductal carcinoma in situ (DCIS) (N = 32) and 5: Invasive ductal carcinoma (IDC) (N = 28).

**Figure 2:**
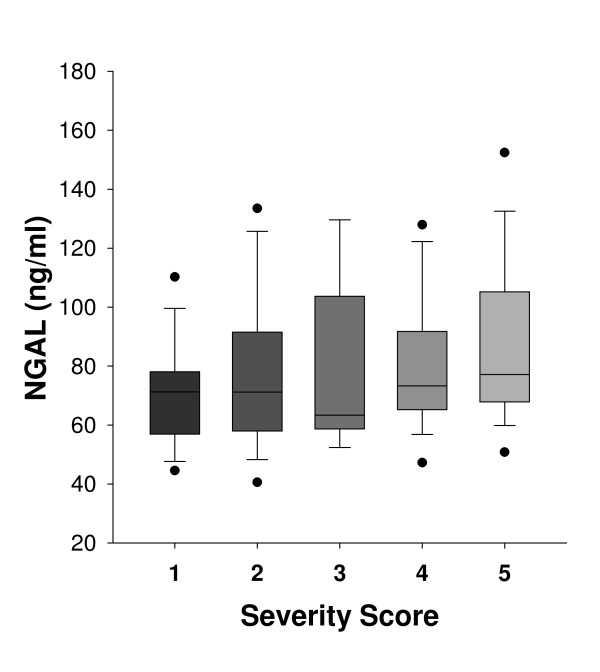
**Serum levels of NGAL depicted as box-plots**. (Boxes represent the interquartile range; lines inside boxes represent the median value; whiskers represent 5^th ^and 95^th ^percentiles). The 1-5 scale represents breast abnormalities of increasing severity as follows: 1: Healthy controls (N = 30), 2: Sclerosing adenosis (N = 35), 3: Atypical ductal hyperplasia (ADH) (N = 18), 4: Ductal carcinoma in situ (DCIS) (N = 32) and 5: Invasive ductal carcinoma (IDC) (N = 28).

**Figure 3:**
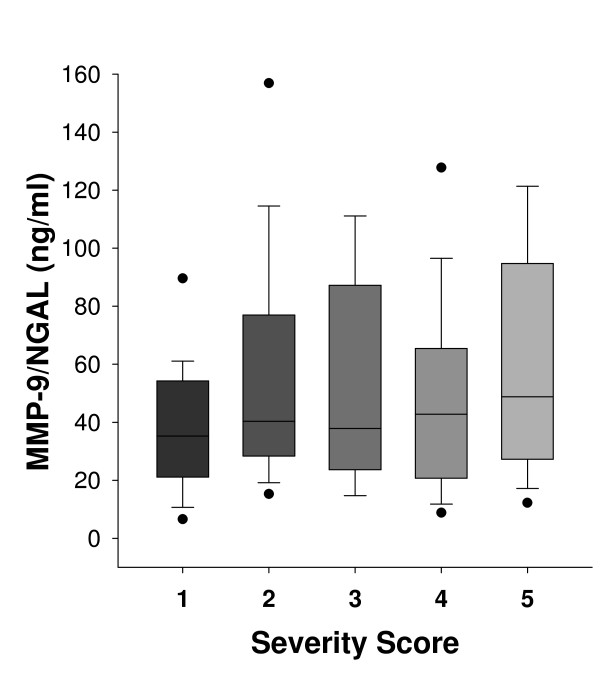
**Serum levels of MMP-9/NGAL complex depicted as box-plots**. (Boxes represent the interquartile range; lines inside boxes represent the median value; whiskers represent 5^th ^and 95^th ^percentiles). The 1-5 scale represents breast abnormalities of increasing severity as follows: 1: Healthy controls (N = 30), 2: Sclerosing adenosis (N = 35), 3: Atypical ductal hyperplasia (ADH) (N = 18), 4: Ductal carcinoma in situ (DCIS) (N = 32) and 5: Invasive ductal carcinoma (IDC) (N = 28).

Increased MMP-9 serum levels were observed for all patients compared to healthy subjects (Figure 1). It is noteworthy that women with sclerosing adenosis exhibited significantly elevated MMP-9 expression, similar to that observed for invasive carcinoma (Table 1). A significant positive correlation was observed between MMP-9 serum levels and disease severity score (r = 0.229, p < 0.006). Regarding NGAL, increased serum expression was observed for patients with breast disease compared to healthy controls (Figure 2). NGAL serum levels were similar between women with DCIS, ADH and sclerosing adenosis whereas a marked increase was observed for women with invasive carcinoma (Table 1). A significant positive correlation between NGAL serum levels and disease severity score was found (r = 0.206, p < 0.02). Higher serum levels of MMP-9/NGAL were observed in IDC, DCIS, ADH and sclerosing adenosis patients compared to healthy women (Figure 3). Increased expression was mainly observed for patients with invasive carcinoma, whereas DCIS, ADH and sclerosing adenosis patients exhibited similar expression levels (Table 1). A positive non-significant correlation was found between MMP-9/NGAL complex expression and disease severity score (r = 0.123, p > 0.14). MMP-9, NGAL and MMP-9/NGAL complex serum levels were not significantly correlated with either age or BMI of patients (p > 0.1).

## Discussion

NGAL appears to protect MMP-9 from autodegradation, increasing its activity by binding and forming MMP-9/NGAL complexes. Tumor cells excrete elevated levels of NGAL resulting in an increase in local concentration of MMP-9, which can affect various aspects of tumor progression [22]. NGAL is abundantly expressed in adipose tissue and liver and recent studies have correlated circulating NGAL levels with obesity and its metabolic complications [32,33]. NGAL has also been suggested as a marker of acute kidney injury [6-9]. Therefore normal liver and renal function was clinically confirmed for all patients included in this study prior to their enrollment. Moreover, no patients with metabolic syndromes participated in the study. According to our findings, serum levels of MMP-9, NGAL, and MMP-9/NGAL were not associated with obesity since no correlation with patients' BMI was observed.

Immunohistochemical studies on breast tissue have associated MMP-9 expression with a higher rate of distant metastases [34-36]. The prevention of the metastatic process is the main aim of the clinicians. Soluble biomarkers could be useful tools in the prediction of patient outcome and management of the disease. Therefore, MMP-9 detection in sera could provide significant information of the tumor biological features. Somiari and coworkers have suggested that circulating MMP-2 and MMP-9 levels are associated with disease severity and may permit the classification of patients with breast disease [24,37]. These findings were recently extended from Wu et al, who reported that serum MMP-9 levels were significantly elevated in patients with breast cancer compared to those with benign breast disease and healthy controls. Additionally, increased MMP-9 levels were associated with lymph node metastasis, higher tumor stage, lower relapse-free and overall survival [36]. In agreement with these observations, our study reported an increase in MMP-9 expression in patients with ADH, DCIS and IDC (p < 0.003) compared to healthy controls. Our results also document a positive correlation between MMP-9 serum levels and the severity score of breast disease (p < 0.006).

Recent evidence suggests that NGAL expression is associated with cancer invasive progression. Gene expression profiling and analysis of human pancreatic adenocarcinomas by cDNA microarrays, quantitative real-time RT-PCR and immunohistochemistry demonstrated increased expression of NGAL in malignant pancreatic tissue compared to normal [38]. Lim et al reported that tissue expression of NGAL in ovarian tumors changes with disease grade and this is also reflected in serum levels [17]. More specifically, tissue NGAL expression was undetectable in normal ovaries, weak to moderate in benign tissues, while it displayed highest levels in borderline and low-grade tumors. The authors also reported similar findings for NGAL expression in serum, with levels being significantly higher in patients with benign and grade 1 tumors compared to healthy controls. The role of NGAL was also investigated in esophageal squamous cell carcinoma (ESCC) and it was reported that its tissue expression was significantly higher in ESCC than in normal mucosa, and was positively correlated with cell differentiation [13]. Based on their findings that NGAL in human tissue and urine samples were consistently associated with invasive breast cancer, Yang et al suggested that NGAL may be a potential noninvasive biomarker of breast disease [39]. Our findings further support the hypothesis that NGAL is associated with cancer disease severity. Serum NGAL levels were higher in patients with IDC (p < 0.008) while moderate in patients with DCIS, ADH and sclerosing adenosis compared to healthy controls. A significant positive correlation between NGAL expression and breast disease severity score was also observed (p < 0.02).

Regarding MMP-9/NGAL, current evidence suggests that urinary detection of the complex may represent a new biomarker for the prediction of cancer disease [25,26]. NGAL overexpression in human breast cancer cell lines was accompanied by increased tumor growth, MMP-9 activity, angiogenesis and cell proliferation. Moreover, MMP-9/NGAL enzymatic activity was observed in the urine of breast cancer patients but not in healthy controls [25]. Evaluation of MMP-9 and MMP-9/NGAL complex in urine of patients with brain tumors revealed significantly higher expression levels compared to controls, which was also confirmed in tumor tissue. After tumor resection, clearing of biomarkers was observed. The findings support an association between MMP-9 and MMP-9/NGAL urine levels with the presence of disease and response to therapy [26]. In our study, we attempted to evaluate the expression of MMP-9/NGAL complex in serum of patients with breast disease and correlate it with disease severity. Despite observing higher levels of MMP-9/NGAL in IDC (p < 0.01), DCIS, ADH and sclerosing adenosis patients compared to healthy controls, a positive non-significant correlation between complex expression and disease severity score was found. However, future studies including a higher number of patients could elucidate the significance of serum MMP-9/NGAL expression in patients with breast disease.

The relation between breast benign diseases and invasive cancer has been a matter of discussion for many years, as these lesions are considered precursors in malignant transformation. Sclerosing adenosis is a benign proliferative lesion without atypia with a relative risk for malignant breast disease of 1.3-1.9, while ADH has a slightly higher risk (× 3.9-5.0) [31]. Since sclerosing adenosis and ADH are believed to constitute distinct precursors of invasive breast carcinoma, we attempted to investigate potential differences in the tested parameters. Higher levels of NGAL and MMP-9/NGAL complex were observed for both groups of patients compared to healthy controls, whereas MMP-9 expression was particularly elevated in the sclerosing adenosis group. This observation seems to support the idea that this lesion is a distinct entity of benign disease. Whether serum levels of MMP-9, NGAL and their complex can reflect the relative risk of patients with benign diseases for developing breast cancer remains to be investigated in future studies.

Improved non-operative diagnostic techniques enable the detection of breast cancer at an earlier stage. At the same time, serum measurements represent a non-invasive, easily accessible method for the study of biomarkers as screening tools for risk assessment, diagnosis and prognosis of breast cancer. The present study was designed in an effort to reveal specific biomarkers associated with breast disease progression. Our data suggest that MMP-9 and NGAL are positively correlated with breast disease severity, with a potential clinical utility as early markers of breast disease status.

## Conclusion

In spite of the supporting evidence in the literature, the significance of circulating MMP-9 in prognosis and progression of breast cancer disease requires further clarification. On the other hand, NGAL and MMP-9/NGAL complex have not yet been evaluated in breast cancer. Even though the detection of NGAL and MMP-9/NGAL complex in systemic circulation is likely to directly reflect their tissue expression, scarce data are currently available on the significance of their serum measurement in cancer disease. To our knowledge, this is the first study attempting to investigate the serum expression of MMP-9, NGAL and MMP-9/NGAL complex during diagnostic work-up of patients with breast abnormalities and correlate it with the severity of the disease. Our findings suggest that both MMP-9 and NGAL serum levels are correlated with breast cancer progression since they seem to follow a gradual increase with disease severity, supporting their potential role as early biomarkers.

## Competing interests

The authors declare that they have no competing interests.

## Authors' contributions

XP (Ph.D.) performed the experimental analysis and interpretation of the data and wrote the article. AG (Ph.D.) was responsible for the conception and design of the study, the revision of the article and its final approval. EK (BSc) performed the experimental analysis and interpretation of the data. FZ (MD) and IF (MD) acquired patients' characteristics and samples. AN (MD) performed the immunohistochemical analysis of the biopsies. IP (Ph.D.) and EG (MD) performed the statistical analysis, data interpretation and edited the article. GZ (MD, FACS) performed the VABB biopsies and edited the article. All authors read and approved the final manuscript.

## Pre-publication history

The pre-publication history for this paper can be accessed here:

http://www.biomedcentral.com/1471-2407/9/390/prepub
